# Factors associated with delayed uptake of cataract surgery among adult patients at Mulago National Referral Hospital, Uganda

**DOI:** 10.4314/ahs.v21i3.36

**Published:** 2021-09

**Authors:** Francis O Sebabi, Walter O Okello, Faith Nakubulwa, Rogers Sempindu, Catherine Driciru, Caroline Nalukenge, Ben Mulinde, Lydia Nakiyingi, Damalie Nakanjako, Grace N Ssali, Anne A Musika

**Affiliations:** 1 Department of Ophthalmology, College of Health Sciences, Makerere University, Kampala, Uganda; 2 Department of Medicine, College of Health Sciences, Makerere University, Kampala, Uganda; 3 ChildFund International, Kampala, Uganda

**Keywords:** Risk factors, operable cataract, cataract, surgery, Uganda, sub-Saharan Africa

## Abstract

**Background:**

Cataract is the leading cause of blindness globally. Many patients with cataract in developing countries delay to come for cataract surgery.

**Objectives:**

This study aimed to determine the factors associated with delayed uptake of cataract surgery among adult patients seen at Mulago National Referral Hospital eye clinic in Uganda.

**Methods:**

Employing a hospital based cross-sectional study, adult patients with cataract and having moderate visual impairment or blindness were recruited. Patient-related factors for delayed surgery were assessed using a predetermined questionnaire. Data was analyzed using stata version 14.2. Logistic regressions were used to determine the factors associated with delayed uptake of cataract surgery among these patients.

**Results:**

Eighty two participants with operable cataract were evaluated. Females were 44 (54%) and the mean age of participants was 67 years. Fifty three (65%) had delayed uptake of cataract surgery. The factors associated with delayed uptake of cataract surgery among patients with cataract were financial constraint, felt no need for surgery and good unilateral vision.

**Conclusions:**

Financial constraints, no felt need for cataract surgery and having good unilateral vision are the factors associated with delayed uptake of cataract surgery among cataract patients. We recommend cataract surgical outreach to remote areas and health education.

## Introduction

Cataract is a leading cause of blindness globally^1^. Additional one million people develop cataract blindness annually. In developing countries, cataract causes 51% of blindness compared to 5% in developed counterparts^1^,^2^. Cataractblindness is more in Africa and parts of China^1^, ^2^. Patients presenting for cataract surgery when the eye involved is already blind (Visual Acuity less than 3/60) is described as having delayed uptake of cataract surgery. In sub-Saharan Africa, delayed or no uptake of cataract surgery causes the majority of blindness.

Cataract surgery is the only definitive treatment modality for cataract blindness. Unless cataract surgery is done earlier, the patient has an extended stay with blindness and requires an able person for help. The cataract surgical rate (CSR) for Africa stands at 300 per million population per year. The WHO recommends a CSR of 2,000 per million populations for Africa^2^. Some studies cited cost, fear of surgery, and long distances to the hospital as causes of delayed uptake of cataract surgery^2^, ^3^.

Currently, approximately 42,000 Ugandans have cataract blindness^4^, ^5^. Surgery treats cataract blindness and is available in Uganda^2^. The reasons why the patients with cataract delay to seek cataract surgery resulting to high number of blindness are not known. We evaluated the factors associated with delayed uptake of cataract surgery among adult patients with operable cataract at Mulago National Referral Hospital eye clinic in Uganda.

## Material and methods

### Design and setting

This was a hospital based cross section study. Conducted at Mulago National Referral Hospital eye clinic between February 2020 and March 2020. Mulago National Referral Hospital is located approximately 5 kilometers in the northern part of Kampala city and provides in-patient and out-patient services for patients in Kampala and its surrounding districts in Uganda. Ophthalmology is one of departments in the hospital. The department provides both specialized and general outpatient and in-patient ophthalmic services. Each day, about 40–50 ophthalmic outpatients receive their appropriate care services from the department. Out ofthese, an average of five adult patients present with operable cataract and an average seven cataract cases are seen at the clinic. Some patients have bilateral cataracts.

Ethical clearance for this study was obtained from School of Medicine Research and Ethics Committee (SOMREC) of Makerere University. All the patients received full explanation using English and local languages about purpose and procedures of the study prior to their voluntary written consent for enrollment.

### Participant enrolment and procedures

All adult patients who attended the eye clinic during the period of February and March 2020 were screened for moderate visual impairment (VA<6/18) of either or both eyes. Patients with moderate visual impairment or worse were then assessed for cataract. We included all adult patients with cataract and moderate visual impairment or worse. No patient was excluded.

A consultant Ophthalmologist evaluated all the patients and made diagnosis of cataract using slit lamp biomicroscopy. A trained Ophthalmic Nursing Officer assessed distance visual acuity using Optotype E-chart, conducted the interview and completed the standardized questionnaires. The participants' data such as gender, age, education status, distance from Mulago hospital and residence were collected. Data on factors limiting uptake of cataract surgery such as financial constraint, other ocular morbidities, diabetes mellitus, hypertension, lacking transport money, felt no need for surgery, ability to see with one eye and presence of cataract on both eyes among others were also obtained from the participants. Uniformity and reliability of the data was ensured by using the same trained Ophthalmic Nursing Officer to administer all the questionnaires and the patients were clinically evaluated by the same consultant Ophthalmologist. The questionnaire was pretested one week before the actual data collection and was accordingly modified.

### Data management and statistics

The data was summarized using mean, frequencies and percentages. Stata version 14.2 was used for the statistical analyses. Pearson's Chi correlation, bivariate and multivariate logistic regressions were used to determine factors associated with delayed uptake of cataract surgery. The p<0.05 was considered statistically significant in Pearson's Chi-square correlation, bivariate and multivariate logistic regressions.

## Results

### Demographic characteristics of the adult patients with operable cataract

Eighty two (82) patients with operable cataract were evaluated. Forty four (54%) were females. The mean age of participants was 67 years and 53 (65%) had delayed uptake of cataract surgery and presented with visual acuity of less than 3/60 on either eye or both eyes. The patients of age more 50 years constituted the majority with 53 (65%) and those from the age of 18 -35 years and 36–50 years shared the remaining proportion with nearly equal proportions of 15 (18%) and 14 (17%) respectively. Twenty (24%) had no formal education. This demographic information is shown in table 1.

**Table 1:** Demographic characteristics of the adult patients with operable cataract who attended Mulago National Referral Hospital eye clinic (n=82)

Patients Characteristics	Frequency (%)	DU (N=53)	NDU (N=29)	P value
		No. (%)	No. (%)	
**Gender**				
Male	38 (46)	28 (53)	10 (34)	
Female	44 (54)	25 (47)	19 (66)	0.114
**Age**				
18–35 years	15 (18)	14 (26)	1 (3)	
36–50 years	14 (17)	8 (15)	6 (21)	**0.044** *
More than 50 years	53 (65)	31 (58)	22 (76)	**0.032** *
**Education**				
Illiterate	20 (24)	13 (25)	7 (24)	
Primary	30 (37)	20 (38)	10 (34)	0.903
O level	18 (22)	13 (25)	5 (17)	0.633
A level / certificate	7 (9)	5 (9)	2 (7)	0.757
Diploma and above	7 (9)	2 (4)	5 (17)	0.109
**Distance from MNRH**				
Within 5km	14 (17)	11 (21)	3 (10)	
More than 5km	68 (83)	42 (79)	26 (90)	0.240
**Residence**				
Live in urban	38 (46)	22 (42)	16 (55)	
Live in rural	44 (54)	31 (58)	13 (45)	0.237
**District**				
Kampala	20 (24)	14 (26)	6 (21)	
Wakiso	31 (38)	20 (38)	11 (38)	0.685
Others	31 (38)	19 (36)	12 (41)	0.526

* denotes statistical significance at p-value <0.05 and conf. interval of 95%, DU=Delayed Uptake of cataract surgery, NDU=No Delayed Uptake of cataract surgery

### Patient-related factors for delayed uptake of cataract surgery

The patient-related factors such as to financial constraint, lack of transport money, need for cataract surgery not felt, ability to see with one eye, having diabetes mellitus or hypertension, reduction of vision on one eye or both eyes and having cataract on one or both eyes were assessed for association to delayed uptake of cataract surgery as shown in table 2.

**Table 2:** Patient-related factors for delayed uptake of cataract surgery among adult patients with operable cataract who attended Mulago National Referral Hospital eye clinic (n=82)

Factors	Frequency (%)	DU N=53 (%)	NDU N=29 (%)	P=value
**Financial constraints**				
Constrained	45 (55)	37 (70)	8 (28)	**0.000** *
Not Constrained	37 (45)	16 (30)	21 (72)	
**No transport money**				
Yes	47 (57)	37 (70)	10 (34)	
No	35 (43)	16 (30)	19 (66)	**0.003** *
**Needs not felt**				
Yes	12 (15)	3 (6)	9 (31)	
No	70 (85)	50 (94)	20 (69)	**0.005** *
**Able to see with one eye**				
Yes	12 (15)	11 (21)	1 (3)	
No	70 (85)	42 (79)	28 (97)	**0.063** **
**Diabetic**				
Yes	14(17)	4 (7)	10 (34)	**0.004** *
No	68(83)	49 (93)	19 (66)	
**Hypertensive**				
Yes	12(15)	3 (6)	9 (31)	**0.005** *
No	70 (85)	50 (94)	20 (69)	
**Visual status**				
Reduced vision-unilateral	34 (41)	28 (53)	6 (21)	
Reduced vision-bilateral	48 (59)	25 (47)	23 (79)	**0.006** *
**Laterality of cataract**				
Bilateral	55 (67)	30 (57)	25 (86)	**0.010** *
Unilateral	27 (33)	23 (43)	4 (14)	

* denotes statistical significance at p-value <0.05 and conf. interval of 95%, DU=Delayed Uptake of cataract surgery, NDU=No Delayed Uptake of cataract surgery

** p value <0.05 was from Pearson's Chi-square correlation.

### Visual acuity of the study participants

Twenty six (32%) participants had right eye visual acuity (VA) of less than 3/60 and 38 (46%) of the participants had left eye VA of less than 3/60. Eleven (13%) of the participants had VA less than 3/60 on both eyes.

Factors associated with delayed uptake of cataract surgery among adult patients with operable cataract

In the bivariate logistic regression, patient's age ranging from 36–50 years, patient's age greater than 50 years, financial constraint, diabetes mellitus, hypertension, having transport money, feeling no need for cataract surgery, ability to see with one eye and having bilateral cataract were all statistically significantly associated with delayed uptake of cataract surgery as shown in table 1. The multivariate logistic regression found that financial constraints (aOR 3.877 p=0.023), lack of felt need for cataract surgery (aOR 19.713 p=0.019) and ability to see with one normal eye (aOR 0.026 p=0.011) were the factors statistically significantly associated with delayed uptake of cataract surgery as shown in table 3.

**Table 3:** Factors associated with delayed uptake of cataract among adult patients with operable cataract who attended Mulago National Referral Hospital eye clinic (*N*=82)

Factors	cOR (95%CI)	p-value	aOR (95% CI)	p-value
Age from 36–50 years	0.095 (0.010–0.939)	**0.044** *	0.265 (0.016 – 4.32)	0.351
Age ≥50 years	0.101 (0.012–0.823)	**0.032** *	0.351 (0.028 – 4.369)	0.416
Financial constraint	6.070 (2.225 – 16.559)	**0.000** *	**6.126 (1.117 – 33.586)**	**0.037** *
Diabetes Mellitus	0.155(0.043 – 0.555)	**0.004** *	0.359 (0.053 – 2.434)	0.294
Hypertensive	0.133(0.033 – 0.544)	**0.005** *	0.309 (0.033 – 2.895)	0.303
Had transport money	0.228 (0.087 – 0.597)	**0.003** *	2.256 (0.372–13.696)	0.377
No Need for surgery	7.500 (1.839 – 30.590)	**0.005** *	**19.713 (1.640 – 237.000)**	**0.019** *
Able to see with an eye	0.136 (0.017 – 1.116)	**0.063** **	**0.026 (0.002 – 0.432)**	**0.011** *
Bilateral cataract	4.792 (1.462 – 15.704)	**0.010** *	2.324 (0.361 – 14.975)	0.375

* denotes statistical significance at p-value <0.05 and conf. interval of 95%

** p value <0.05 was from Pearson's Chi-square correlation, cOR=crude Odd Ratio, 95%CI Confidence Interval

Factors such as patient's age of 36–50 years (aOR 0.26 p=0.351) and more than 50 years (aOR 0.351 p=0.416), diabetes mellitus (aOR 0.359 p=0.294), hypertension (aOR 0.309 p=0.303), having transport money (aOR 2.256 p=0.377) and having unilateral cataract (aOR 2.324 p=0.375) were not statistically significantly associated with delayed up take of cataract surgery as indicated in table 3.

## Discussion

Our study found that 65% of patients with operable cataract who attended Mulago National Referral Hospital eye clinic had delayed uptake of cataract surgery and the factor for delayed uptake of cataract surgery were financial constraints, lack of felt need for surgery and having ability to see with one eye.

A study conducted among walk-in patients with operable cataract in India reported that 42% had delayed uptake of cataract surgery and presented for cataract surgery with bilateral visual acuity of less than 3/60^6^. Unlike the study in India, our study included patients with unilateral blindness (VA<3/60) in the categorization of delayed uptake of cataract surgery. This is thought to have increased the prevalence of delayed uptake of cataract surgery among the patients with operable cataract in our study.

In assessing the factors associated with delayed uptake of cataract surgery, our study found that financial constraint was positively statistically significantly associated with delayed uptake of cataract surgery. More specifically, the adult patients with operable cataract who experienced financial constraints were six times more likely to have delayed uptake of cataract surgery than those who were not constrained. This finding is compared to findings in Nigeria, Gambia, Nepal and India stating that cost of operations, transport, feeding and escort hindered timely uptake of cataract surgery^7^–^10^. In similar setting in Grarbet Eye Hospital in Ethiopia, financial constraints was found to hinder uptake of cataract surgery even if the actual surgery cost is subsided^2^. This finding therefore implies that, money is still required for enabling utilization of cataract surgery even if direct cost is minimized. The money is needed for transport, feeding and accompanying attendant. Other indirect costs of utilizing cataract surgery could include loss of daily income and delegating household responsibilities. Reducing direct and indirect cost can be done by taking the services nearer to where patient with cataract stays; conducting high volume cataract surgery and bulk purchasing of consumables. These allow even the poorest segment of the population to receive ophthalmic care and improves uptake of cataract surgery.

The study further found that patients' perception of lack of felt need for cataract surgery was associated with delayed uptake of cataract surgery. A patient with operable cataract who did not feel the need for cataract surgery was 20 times likely to have delayed uptake of cataract surgery than those felt that they needed it. This finding is similar to a result from in South India. Patients who lacked felt need for cataract surgery would delay to utilize cataract surgical services^11^. Finding during the Rapid Assessment of Avoidable Blindness (RAAB) in mid-western Uganda also concluded that patients with operable cataract who did not feel the need for the operation did not come timely for cataract surgery^9^. This implies that negative attitude impedes utilization of cataract surgery. One of the ways of overcoming effect of negative attitude towards health care utilization is health education.

Participants with good vision on one eye had less likelihood to have delayed uptake of cataract surgery. The results of this study contrast with other studies that have already revealed positive associations between ability to see with one eye and delayed uptake of cataract surgery among adult with operable cataract. Findings from rural China indicated that failure to understand the need of cataract surgery is one of the factors influencing uptake of cataract surgery^10^. One would not find the need for surgery because of being able to see with the other eye. In some studies, good vision in the one normal eye makes the patient with cataract to delay coming for surgery^7^. The finding among the adult patients with operable cataract in MNRH also contrasts with a study in Kenya that established that people with better vision were more likely to delay uptake of cataract surgery than those with poorer vision^12^. This finding therefore implies that patients with ability to see with one normal eye enables them to come to hospital seeking cataract surgery timely. This denotes good eye health practices among the population who get eye health services. On the hand, it may indicate that patients who are bilaterally blind may not be able to reach the Mulago National Referral Hospital for cataract surgery. These findings therefore require addition study to establish the reasons as to why patients with unilateral vision come more timely compared to those with no vision completely.

This study being a hospital based, it has limited generalizability to the individuals with operable cataract who did not present at Mulago Hospital for clinical evaluation. There are limitations resulting from using severity of the visual loss (VA<3/60) to equate to delayed uptake of cataract surgery instead of duration of time to measure delayed uptake of cataract surgery.

## Conclusion

A large proportion of patients with operable cataract at the Mulago National Referral Hospital eye clinic have delayed uptake of cataract surgery and the factor for delayed uptake of cataract surgery were financial constraints, lack of felt need for cataract surgery and having ability to see with one eye. We recommend Cataract surgical outreach that take services to rural areas to minimize financial requirements for surgery. We also recommend health education to increase awareness on the need for surgery to prevent cataract blindness.

## References

[1] de Almeida Ferreira G, Schaal LF, Ferro MD, Rodrigues ACL, Khandekar R, Schellini SA (2017). Outcomes of and barriers to cataract surgery in Sao Paulo State, Brazil. BMC Ophthalmology.

[2] Mehari ZA, Zewedu RTH, Gulilat FB (2013). Barriers to cataract surgical uptake in central ethiopia. Middle East African Journal of Ophthalmology.

[3] Mpyet C, Dineen B, Solomon A (2005). Cataract surgical coverage and barriers to uptake of cataract surgery in leprosy villages of north eastern Nigeria. British Journal of Ophthalmology.

[4] Bastawrous A, Dean WH, Sherwin JC (2013). Blindness and visual impairment due to age-related cataract in sub-Saharan Africa: a systematic review of recent population-based studies. British Journal of Ophthalmology.

[5] Uganda Buraeu of Statistics Uganda Beraeu of Statistics (2020). Enhancing Data Quality and Use.

[6] Khandekar R, Sudhan A, Jain B, Deshpande M, Dole K, Shah M (2015). Impact of cataract surgery in reducing visual impairment: a review. Middle East African Journal of Ophthalmology.

[7] Najmul Aqib Khan M, Ansari MA, Ahmad A, Khalil S, Maroof M (2017). A study to assess the barriers for cataract surgery uptake among elderly population of Aligarh.

[8] Ocansey S, Kyei S, Gyedu BN, Awuah A (2014). Eye care seeking behaviour: a study of the people of Cape Coast Metropolis of Ghana. J Behav Health.

[9] Kikira S, Kasadhakawo M, Bakaki W, Binta J (2013). RAAB IN HOIMA.

[10] Zhang XJ, Jhanji V, Leung CK-S, Li EY, Liu Y, Zheng C (2014). Barriers for poor cataract surgery uptake among patients with operable cataract in a program of outreach screening and low-cost surgery in rural China. Ophthalmic Epidemiology.

[11] Kovai V, Krishnaiah S, Shamanna BR, Thomas R, Rao GN (2007). Barriers to accessing eye care services among visually impaired populations in rural Andhra Pradesh, South India. Indian Journal of Ophthalmology.

[12] Briesen S, Roberts H, Ilako D, Karimurio J, Courtright P (2010). Are blind people more likely to accept free cataract surgery? A study of vision-related quality of life and visual acuity in Kenya. Ophthalmic epidemiology.

